# Authors' Response to Gowdy

**DOI:** 10.1371/journal.pmed.0030414

**Published:** 2006-09-26

**Authors:** Atle Fretheim, Andrew D Oxman

We thank A. D. Gowdy for his comments [1] on our article [2]. He suggests that the National Health Service and other health-care providers have a lot to learn from the pharmaceutical industry. Where is the evidence?

We are not aware of data that convincingly demonstrate the impact of outreach visits by pharmaceutical representatives. Gowdy indicates that such information exists (“Progress is tracked meticulously”). We would very much like to see it!

We have had informal discussions with executives from companies in Norway, and we have been struck by how they themselves question the effectiveness of their marketing strategies. At a recent conference in Denmark, the medical director of a major pharmaceutical company gave a talk on the impact of industry marketing on prescribing habits [3]. He had no other data to show than a handful of anecdotes, and when questioned about this he insisted that neither he nor his marketing department was aware of more rigorous evaluations.

The degree of interaction between the pharmaceutical industry and the medical profession is associated with differences in prescribing patterns [4]. Thus, what the pharmaceutical industry is doing in terms of marketing does seem to work, at least to some extent. However, the marketing effort made by industry is massive and includes a wide range of interventions. It is difficult to know what the relative merit of each component is.

Even more difficult to estimate is the cost-effectiveness of various marketing strategies. Considering that the pharmaceutical industry spends a five-digit amount ($US) per doctor per year on marketing alone [4], the industry should achieve substantial effects to compare favourably with, for instance, our results: We spent $US500 per doctor and achieved a doubling of thiazide prescriptions [5].

The only study cited by Gowdy did indeed show promising results. However, changes in prescribing were compared between practices that chose to participate in the programme and practices that chose not to [6], and whether this is a fair comparison is uncertain. Moreover, he does not put this study into the context of a systematic review of the relevant research.

Gowdy thinks our intervention sounds like “a policing approach”. This does not fit with our perception. The doctors were satisfied with the chance of meeting an industry-independent source of information and appreciated the opportunity to reflect on their own practice in light of the information and feedback that we provided them.

Gowdy’s use of the term “evidence-based” when describing the messages conveyed by pharmaceutical companies begs a brief comment. Several investigators have assessed the quality of advertisements and promotional material distributed by the pharmaceutical industry. They consistently conclude with a word of caution against basing clinical practice on claims made by pharmaceutical companies [7–10].
